# Post-marketing surveillance in the published medical and grey literature for percutaneous transluminal coronary angioplasty catheters: a systematic review

**DOI:** 10.1186/2046-4053-2-94

**Published:** 2013-10-10

**Authors:** Julie Polisena, Alan J Forster, Karen Cimon, Danielle Rabb

**Affiliations:** 1 Canadian Agency for Drugs and Technologies in Health, 600-865 Carling Ave, Ottawa, Ontario, K1S 5S8, Canada; 2 Department of Epidemiology and Community Medicine and Epidemiology, University of Ottawa, Ottawa, Ontario, Canada; 3 Clinical Epidemiology Program, Ontario Hospital Research Institute, Ottawa, Ontario, Canada; 4 Performance Management, The Ottawa Hospital, Ottawa, Ontario, Canada; 5 Department of Medicine, Faculty of Medicine, University of Ottawa, Ottawa, Ontario, Canada; 6 Institute for Clinical Evaluative Sciences, Ottawa, Ontario, Canada

**Keywords:** Post-marketing surveillance, Medical device, Incident, Adverse event, Malfunction

## Abstract

**Background:**

Post-marketing surveillance (PMS) may identify rare serious incidents or adverse events due to the long-term use of a medical device, which was not captured in the pre-market process. Percutaneous transluminal coronary angioplasty (PTCA) is a non-surgical procedure that uses a balloon-tipped catheter to enlarge a narrowed artery. In 2011, 1,942 adverse event reports related to the use of PTCA catheters were submitted to the FDA by the manufacturers, an increase from the 883 reported in 2008. The primary research objective is to conduct a systematic review of the published and grey literature published between 2007 and 2012 for the frequency of incidents, adverse events and malfunctions associated with the use of PTCA catheters in patients with coronary artery disease (CAD). Grey literature has not been commercially published.

**Methods:**

We searched MEDLINE, EMBASE, the Cochrane Central Register of Controlled Trials and PubMed for medical literature on PMS for PTCA catheters in patients with CAD published between January 2007 and July 2012. We also searched the grey literature.

**Results:**

This review included 11 studies. The in-hospital adverse events reported were individual cases of myocardial infarction and hematoma. In studies of patients with coronary perforation, more patients with balloon angioplasty were identified compared with patients who required stenting.

**Conclusions:**

Our systematic review illustrates that the volume and quality of PMS studies associated with the use of PTCA catheters in patients with CAD are low in the published and grey literature, and may not be useful sources of information for decisions on safety. In most studies, the objectives were not to monitor the long-term safety of the use of PTCA catheters in clinical practice. Future studies can explore the strengths and limitations of PMS databases administered by regulatory authorities.

## Background

The US Food and Drug Administration (FDA) defines a medical device as an instrument used to diagnose, treat or prevent a disease or abnormal physical condition without any chemical action in the body [1]. The medical device industry has grown in the past ten years. Between 2004 and 2009, the sales of medical devices increased by 56%, while pharmaceutical sales increased by 38% during the same period [2]. Excluding diagnostics, the medical device industry is a USD 200 billion business worldwide, with projected sales of USD 95 billion in 2010 in the US alone [2].

Unlike drug therapies, the approval processes for medical devices typically do not require clinical effectiveness and safety data derived from a randomized clinical trial (RCT) [3], even for FDA class III medical devices, which have the highest risk for patients. Manufacturers must perform studies on human subjects, but there are no standards for sample size, design or follow-up period, as there are for medications [4,5]. Thousands of medical device applications are submitted each year in the US. However, fewer than 100 were considered to be high-risk devices and went through a pre-market approval (PMA) process. Instead, most applications undergo a 510(k), a pre-market submission required by the FDA, where manufacturers claim that their device is as safe and effective as the comparator device available on the market. In these studies, measures of safety and effectiveness are not mandatory [3]. PMA is the strictest submission process and the FDA requires that the submission contains valid scientific evidence to ensure the safety and effectiveness of the medical device during its intended use [6]. Equivalent clinical evidence, therefore, would unlikely be available across all medical devices, rendering product comparisons nearly impossible [7].

Post-marketing surveillance (PMS) and epidemiology programs are complementary to the pre-market process since they may identify rare serious adverse events due to long-term use of the medical device not captured previously [4,8]. PMS collects data from the monitoring and assessment of adverse reactions to marketed health products, as well as standard market intervention and communication procedures, and associated policy development and business transformation activities [9]. Challenges associated with PMS studies include finding sources with relevant medical device data and identifying a patient population that had been exposed to a specific medical device [10]. It is possible that device-related adverse events are underreported since manufacturers are not usually obliged to search for device malfunctions actively [11]. Further, there may be a disincentive to report adverse events or device malfunctions if health-care providers use them in patient populations not originally approved by the regulatory authority [11,12].

Given the above limitations in the regulatory requirements for monitoring the safety of medical devices, it is possible that there are significant deficiencies in their safety. While this might be true for all devices, we have decided to focus specifically on cardiovascular devices. The volume, complexity and costs of cardiovascular devices are on the rise [11,12]. For example, the prevalence of coronary artery disease (CAD) in 2005 was 16 million people in the US, and the estimated direct and indirect cost of CAD for 2008 was USD 156.4 billion [13]. Percutaneous transluminal coronary angioplasty (PTCA) is a non-surgical procedure, which uses a balloon-tipped catheter to enlarge a narrowed artery as an alternative to open-heart surgery [14]. The PTCA catheter was approved by the FDA in 1980. In 2010, the FDA reclassified standard PTCA catheters from class III to class II (that is, lower risk) when used for balloon dilation to treat narrowed or blocked arteries in patients with coronary ischemia. To date, 33 catheters made by 10 manufacturers have been approved, including two devices in 2010 and seven devices in 2011 [15].

In 2011, 1,942 adverse event reports related to the use of PTCA catheters were submitted to the FDA by the manufacturers, an increase from the 883 reported in 2008 [15]. It is unclear if the rise in the number of adverse event reports from 2008 to 2011 was related to reclassification of the device from a class III to II risk level (with a less stringent market approval process) or whether a greater number of PTCA catheters were implanted in patients with CAD during the time period. The patient outcomes for 1,662 adverse events reported in 2011 remain unknown. In 2008, 26 adverse event outcomes were reported versus 50 in 2011, and the most frequent device malfunction reported was material rupture. Between 2009 and 2010, there were two class 1 recalls of PTCAs, POWERSAIL® Coronary Dilation Catheters and AngioSculpt PTCA, due to device malfunctions that could lead to serious adverse events, including air embolism, myocardial infarction (MI) and death [16,17]. Class 1 recalls are the most serious type of recall since they may involve serious injury to or death of the patient [16].

The availability of PMS studies on adverse events and malfunctions for PTCA catheters in patients with CAD in the published medical literature is unknown. In addition, the various methods adopted to monitor the safety of this device following its implementation in clinical settings remain elusive. By performing a systematic review of the literature to identify safety problems associated with the device, we may be able to determine whether the published medical literature is a useful source of information to estimate the safety of PTCA catheters. If we find useful information, then this approach could be adapted to monitor the safety of other devices.

### Objective

The primary research objective is to review systematically the medical and grey literature, published between 2007 and 2012, for post-marketing surveillance studies on the frequency of incidents and malfunctions associated with the use of PTCA catheters in clinical practice among patients with CAD.

## Methods

### Literature search strategy

We identified the published literature by searching the following bibliographic databases: MEDLINE (from 1946) with in-process records and daily updates via Ovid, EMBASE (from 1980) via Ovid, the Cochrane Central Register of Controlled Trials (2012, Issue 1) via Ovid and PubMed. The search strategy consisted of both controlled vocabulary, such as the National Library of Medicine’s MeSH (medical subject headings), and keywords. The main search concepts were percutaneous transluminal coronary angioplasty, heart catheterization and cutting or scoring catheters combined with equipment safety and failure, adverse events, post-marketing surveillance, medical device recalls and withdrawals. The search strategy is given in Additional file 1: Table S1. We limited the search to English and French language documents published between 1 January 2007 and 16 July 2012 to reflect the potential impact of the reclassification of PTCA catheters from a class III to class II risk level by the FDA on patient safety. We identified grey literature (literature that is not commercially published) by searching relevant sections of the Grey Matters checklist [18].

### Selection criteria

The selection criteria include RCTs and non-randomized studies, such as cohort and case–control studies, case series and reports, that presented incidents, adverse events, procedure complications or device malfunctions related to the use of PTCA catheters among adult and paediatric populations. Two reviewers (JP and KC) selected the final articles for inclusion based on an examination of the full publications. Any disagreements between the reviewers were discussed until a consensus was reached. For our systematic review, incidents were defined as events or circumstances that could have or did lead to unintended and/or unnecessary harm to a person, and/or a complaint, loss or damage; and adverse events were defined as an unintended injury caused by medical management rather than by a disease process [19].

Conference abstracts, letters and editorials were excluded. In addition, studies conducted to demonstrate the effectiveness and safety of the devices for market approval were not included in this systematic review.

### Article selection

Two individuals (JP, KC) independently reviewed the titles and abstracts of search results and selected articles for inclusion based on the eligibility criteria. Rather than resolving selection differences, all those selected by at least one reviewer were retrieved since ultimate judgment about inclusion must often be reserved until the full text is examined. If more than one publication described a single study and each presented the same data, the most recent was included. Both reviewers compared findings and resolved differences through discussion, after which one study for each eligible instance was captured for further analysis.

### Data extraction

One reviewer (JP) conducted the data abstraction for all included studies using pre-specified extraction forms, and another reviewer (KC) verified the accuracy of the data extracted from all included reports. Any disagreements between the reviewers were discussed until a consensus was reached.

### Quality assessment of included studies

Two reviewers (JP and KC) critically appraised independently the internal validity of the included studies using checklist tools for RCTs and cohort and case–control studies available on the Scottish Intercollegiate Guidelines Network website. Separate methodology checklists by study design were used to assess the internal validity and overall assessment of the study, including the generalizability. Each tool addresses how well a study meets the different components of the study design that may impact the study findings and conclusions [20].

### Data analysis and synthesis

A formal meta-analysis was not performed since the aim of this systematic review is to identify and present the literature on adverse events and device malfunctions for PTCA catheters reported in PMS studies, rather than to test a hypothesis. Instead, the study design, medical device, patient population, health-care setting, interventions, adverse events and device malfunctions measured in each selected study were reviewed and described individually.

## Results

### Quantity of research available

**Figure 1:**
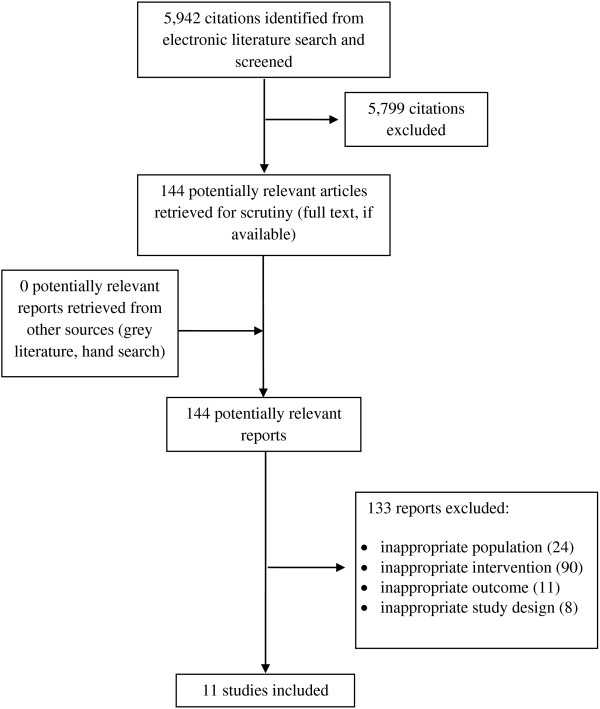
PRISMA flowchart.

The literature search identified 5,942 citations. Of these, the full text of 144 potentially relevant articles was retrieved for further review. For this review, 11 studies were selected for inclusion. Studies were excluded if they included inappropriate study participants, measured an inappropriate device, such as guidewires, guiding catheters and bare-metal or drug-eluting stents, did not specify the devices employed during the percuntaneous coronary intervention (PCI) (also known as coronary angioplasty), measured irrelevant outcomes, or limited the findings to long-term clinical outcomes. The Preferred Reporting Items for Systematic Reviews and Meta-Analyses (PRISMA) flowchart in Figure 1 outlines the study selection process.

### Study characteristics

Four studies were conducted in Germany [21-24], two in Italy [25,26], and one each in Israel [27], Taiwan [28], the US [29] and Japan [30]. One study was performed in Italy and the UK [31]. Table 1 is a complete list of study characteristics.

**Table 1:** Study characteristics

**First author, year and country of publication**	**Study design, length of study, source of funding**	**Population**	**Interventions and comparators**	**Outcomes: adverse events and device malfunctions associated with balloon angioplasty**
Al-Lamee, 2011 [31]	Retrospective case series	56 patients who underwent PCI and had grade III coronary perforation	All patients received aspirin and a loading dose of thienopyridine prior to procedure. All patients then received intravenous heparin with an initial 100-U/kg bolus followed by additional heparin until targeted clotting time >250 s was achieved.	Perforation caused by intracoronary compliant balloon: 15/56; 26.8%
Italy and UK	May 1993 to December 2009			
	Source of funding: NR		PCI was conducted with pre-dilation and stent implantation using standard techniques via the femoral artery. Following the procedure, all patients received aspirin or dual antiplatelet therapy with aspirin and thienopyridine therapy if patient received intracoronary stents.	Perforation caused by intracoronary non-compliant balloon: 13/56; 23.2%
				Perforation caused by intracoronary cutting balloon: 4/56; 7.1%
Werner, 2011 [21]	Single-arm prospective cohort multi-centre study	42 patients with coronary CTOs	All patients were recanalized on aspirin (100 mg) and received clopidogrel (75 mg) with a loading dose of 600 mg for 12 months from the start of procedure.	In-hospital complications in patients with BridgePoint devices:
Germany	July 2008 to June 2009		Lesions were crossed with guidewires (Stingray®, BridgePoint devices) supported by an exchange catheter (CrossBoss™ Catheter, BridgePoint devices) or a low profile over-the-wire catheter (Stingray®, BridgePoint devices).	NSTEMI: 2/42; 4.2%
	Source of funding: NR		Balloon dilation was conducted with increasing sizes, and a stent of an appropriate size was implanted. A balloon-to-artery ratio of 1.1 with inflation pressures of 12 to 16 atm was used.	No penetrations external to adventitia occurred with BridgePoint devices.
				Failure of CrossBoss catheter to cross the proximal cap: 5/18; 27.8%
				Failure of Stingray balloon advancement into proper position: 2/14; 14.3%
				Failure of Stingray balloon advancement after re-entry attempt: 3/10; 30%
				Failure of re-entry puncture because of loss of distal contrast filling: 2/10; 20%
Kandzari, 2011 [29]	Single-arm prospective cohort multicentre study	51 patients with single or multi-vessel CAD	All patients received aspirin (75 to 325 mg/day) before procedure and clopidogrel (75 mg/day for ≥72 hr pre-procedure or ≥300 mg if <72 hr).	There were no occurrences of balloon ruptures or failure of delivery
US	Study period not specified	Patients who intended to undergo treatment of target lesion(s) within native coronary artery or bypass graft with angiographic stenosis ≥70%, including CTOs	Dual antiplatelet therapy and peri-procedural anticoagulation were administered per guideline and protocol recommendations.	Procedural success rate: 51/51 patients; 100%
	Source of funding: several authors received research grants from industry		Device under investigation was a low-profile, zero-fold 1.25-mm-diameter angioplasty balloon (Medtronic CardioVascular, Santa Rosa, CA, US).	Lesion success rate: 54/54 lesions; 100%
			Balloon length, number of catheters and number of inflations used during procedures were up to investigators’ discretion.	
Cortese, 2010 [25]	Single-centre randomized trial	60 adult patients with stable or unstable angina and clinical indication for PCI of at least one small coronary artery (≤2.75 mm)	All patients received aspirin (either 100 mg/day for at least three days prior or pre-PCI 300 mg intravenous bolus), and clopidogrel (300 or 600 mg as a loading dose, followed by 75 mg daily).	Procedural success:
Italy	Two investigators were blinded to patient treatment allocation		28 patients were randomized to Dior paclitaxel-coated balloon (PCB) (Eurocor, Bonn, Germany)	PCB: 27/28; 96.4%
	CTR number (EudraCT code): 2009-012268-15		29 patients were randomized to Taxus™ Liberté™ DES	Taxus stent: 29/29; 100%
				*P* = 0.30
	August 2007 to August 2008			
	Source of funding: NR			
Shimony, 2009 [27]	Case–control study	228 patients who underwent PCI (including wires, balloons and stents)	Group 1: 57 patients with CP	Equipment causing CP:
Israel	January 2001 to December 2008		Group 2: 171 patients without CP	Wire: 30/57; 53%
	Source of funding: NR			Balloon: 15/57; 26%
				Stents: 12/57; 21%
				Equipment causing Grade I CP (Ellis classification):
				Wire: 3/7
				Balloon: 2/7
				Stents: 2/7
				Equipment causing Grade II CP (Ellis classification):
				Wire: 18/30
				Balloon: 7/30
				Stents: 5/30
				Equipment causing Grade III CP (Ellis classification):
				Wire: 9/20
				Balloon: 6/20
				Stents: 5/50
Unverdorben, 2009 [22]	Randomized non-blinded multicentre trial	131 patients for the treatment of coronary in-stent restenosis	All patients were treated with 250 mg of aspirin intravenously, heparin as an initial bolus of 70 to 200 U/kg body weight adjusted according to the activated clotting time with a target of 200 to 250 seconds. One day before the procedure, a loading dose of 300 mg of clopidogrel was administered or 600 mg before the intervention. All patients were assessed based on angiographic inclusion and exclusion criteria.	Success rate with crossing the lesion:
Germany	January 2006 to December 2006		Group 1: 65 patients in DES group (Taxus)	Balloon catheter: 65/65; 100%
	Source of funding: industry		Group 2: 66 patients in coated-balloon catheter group (SeQuent Please)	DES: 61/66; 92.4%
Sardella, 2009 [26]	Three-arm retrospective cohort study	93 patients who underwent DES implantation and subsequently developed DES ISR	57 patients were treated with POBA	No in-hospital adverse events, including death, acute stent thrombosis, myocardial infarction, or new revascularization occurred
Italy	18 months		39 patients were treated with homo-DES implantation (patients eluted with same drug used in previous DES implantation)	
	Source of funding: NR		26 patients were treated with hetero-DES implantation (patients eluted with different drug used in previous DES implantation)	
Chua, 2008 [28]	Single-arm retrospective cohort study	21 patients with CAD who underwent PCI and experienced procedure-related CP	All patients received intravenous heparin (100 units/kg) at the beginning of the procedure.	Occurrence of CP:
Taiwan	October 1992 to December 2006		All patients were treated with percutaneous transfemoral or transradial approach through an angiography sheath. The standard angioplasty technique was applied.	Guidewire manipulation: 5/21; 20.8%
	Source of funding: NR			Coronary balloon angioplasty: 13/21; 61.9%
				Coronary stenting: 3/21; 14.3%
Saito, 2008 [30]	Single-arm retrospective cohort study	45 patients treated with PCI for CTO lesions of coronary arteries	Two 7-French guiding catheters were used in all patients. Microcatheters were inserted into targeted collateral artery with support from guidewires.	One case of transient ischemia in target region caused by a false spasm of tortuous epicardial collateral artery by guidewire and balloon catheter insertion was reported. No residual ischemia was found.
Japan	January 2006 to January 2007		If microcatheters did not pass into target artery, balloon catheters (Ryujin® -OTW 1.25 mm × 10 mm, Terumo, Lacross® 1.30 mm × 10 mm, Goodman, Japan or Maverick® -OTW 1.50 mm × 15 mm, Boston Scientific, US) were used.	
	Source of funding: NR		If both microcatheters and balloon catheters did not cross, Tornus® catheter (ASAHI Intecc) was used. Following the successful crossing of collateral channels, a Miracle® 3 g guidewire (Miracle 3: ASAHI Intecc) was used.	
Braun, 2007 [23] Germany	Non-blinded randomized multi-centre trial	222 patients with stable or specific categories of unstable angina (negative troponin test) and ischaemia or silent ischaemia due to one or two *de novo* lesions in coronary arteries of less than 2.8 mm in diameter	Group 1: 106 patients were treated with standard angioplasty balloon (PEGASO™, SORIN Biomedica, Italy) of 2.5 mm diameter at 6 atm.	In-hospital MACE (MI):
	Six months		Group 2: 116 patients received a 2.5-mm carbon-coated stent (SYNCRO™, SORIN Biomedica Italy) available in 9, 12, 15 and 19 mm of length with or without pre-dilation with a PEGASO™ balloon	POBA: 1/106; 0.9%
	Source of funding: NR			Stent: 0/116; 0%
				*P* = 0.294
				Other events:
				Hematoma at puncture site:
				POBA: 2/106; 1.9%
				Stent: 0/116; 0%
				Aneurysm at puncture site:
				POBA: 0/106; 0%
				Stent: 1/116; 0.01%
				Pericardial effusion:
				POBA: 0/106; 0%
				Stent: 1/116; 0.01%
				Contrast reaction:
				POBA: 2/106; 1.9%
				Stent: 0/116; 0%
				*P* = 0.347
Völzke, 2007 [24] Germany	Three-arm prospective cohort	996 patients with CAD	CABG Group: 240 patients were administered heparin in a dose of 2 to 3 mg/kg before surgery. Aspirin and coumarin were administered following discharge, as necessary	Mortality:
	Approximately 8.5 years		PTCA Group: 478 patients received heparin (10,000 IU) prior to the procedure. All patients received aspirin and coumarin upon discharge as necessary.	PTCA: 95/478; 19.9%
	Source of funding: government		CABG Group: 278 patients received heparin (10,000 IU). These patients had an unsuccessful PTCA or experienced acute complications during the procedure. Subsequently, they received stent implantation. Patients received aspirin (100 mg o.d.) and ticlopidine (250 mg b.d.). All patients received aspirin and coumarin upon discharge after the first 28 days.	CS: 45/278; 16.2%
				CABG: 53/240; 22.1%
				MACE:
				PTCA: 317/478; 66.3%
				CS: 138/278; 49.6%
				CABG: 80/240; 33.3%
				Incident MI:
				PTCA: 31/478; 6.5%
				CS: 22/278; 8.0%
				CABG: 18/240; 7.5%
				Incident percutaneous TVR:
				PTCA: 210/478; 44.1%
				CS: 83/278; 30.2%
				CABG: 26/240; 10.9%
				Incident operative TVR:
				PTCA: 76/478; 16.0%
				CS: 35/278; 12.7%
				CABG: 1/240; 0.4%

*CAD* coronary artery disease, *CP* coronary artery perforation, *CS* coronary stent, *CTO* coronary total occlusion, *CTR* clinical trial registration, *DES* drug eluting stent, *ISR* in-stent restenosis, *MACE* major adverse cardiac event, *MI* myocardial infarction, *NR* not reported, *NSTEMI* non-ST elevation myocardial infarction, *PCI* percutaneous coronary intervention, *POBA* plain old balloon angioplasty, *PTCA* percutaneous transluminal coronary angioplasty, *TVR* target vessel revascularization.

### Study design

Three studies were non-blinded randomized trials [22,23,25]. A single-arm prospective cohort study design was used in three studies [21,28,29] and one study was a three-arm prospective cohort in design [24]. Three studies were retrospective cases series [31], one was a single-arm retrospective cohort [30], one a three-arm retrospective cohort [26] and one a case–control study [27]. Four studies were multi-centred [21-23,29]. The two- or three-armed studies assessed the comparative effectiveness of PTCA catheters with comparable devices or procedures. The one-armed studies aimed to measure the rates of success or complications for the patient with the use of the device.

### Patient population

Two studies included populations with coronary perforations [28,31] and two studies included patients with restenosis [22,26]. Patients with coronary total obstruction lesions were selected in another two studies [22,26], and patients with stable or unstable angina were included in two studies [23,25]. Two studies encompassed patients with single- or multi-vessel CAD [24,29], and the morbidities of a patient population undergoing PCI was not available in one study [27]. Sample sizes ranged from 21 [28] to 996 [24].

### Intervention and comparators

In most studies, patients were treated with aspirin [21,22,25,29,31], thienopyridine [31], clopidogrel [21,22,25,29], heparin [22,28,31] or dual antiplatelet therapy and procedural anticoagulation [29]. Guidewires and guiding catheters were used in four studies as part of the procedure [21,27,28,30]. Two studies incorporated stent implantation in the study population [28,31], and one study used intracoronary compliant, non-compliant or cutting balloons [31]. A balloon’s compliance refers to its expandability: compliant balloons are flexible and non-compliant balloons are considered to be inflexible [32]. Balloon catheters in the studies were the Stingray balloon catheter [21], Ryujin® [30], Lacross® [30], Maverick® [30], PEGASO™ by SORIN Biomedica [23] and balloon catheters by Medtronic Cardiovascular [29]. The use of paclitaxel-coated balloon catheters mad by Eurocar [29] and coated-balloon catheters by SeQuent Please [22] were also studied. Two studies compared the effectiveness and safety of drug-coated balloon catheters with a drug eluting stent (DES) [22,25], two studies compared the outcomes in patients who were treated with standard balloon angioplasty versus those with DES implantation [23,26]. One prospective study compared the long-term prognosis following the PTCA procedure with coronary stenting and CABG [24]. Details of the interventions were not available in one study [27].

### Quality assessment

A quality assessment of single-arm studies was not performed since these study designs were descriptive, provided limited information and did not test any hypotheses. The methodological quality of six studies was reviewed [22-27]. In the case–control study, the interventional cardiologist, who determined the severity of coronary perforation among the patients, was blinded to the clinical outcomes to reduce the risk of information bias [27]. Although the authors conducted a multiple logistic regression analysis to identify the predictors for coronary perforation, the selection of variables was not discussed, and it was uncertain if potential confounders were adjusted for in the study [27]. An assessment of the retrospective cohort study found that it was unclear if the investigators were blinded to the outcomes or if potential confounders were considered in the study design and analysis [26]. Moreover, the authors did acknowledge that failure to randomize the patient populations may have influenced the study findings [26]. Three randomized trials were not blinded, which increases the risk of information bias for the study outcomes [22,23,25]. In one trial, the two study investigators were blinded to the patient treatment allocation [25]. Two studies did not discuss the percentage or number of patients who dropped out or were lost to follow-up before the study ended [22,23], but three out of 57 patients were lost to follow-up in the PICCOLETO study [25]. In addition, intention-to-treat (ITT) analysis was mentioned in two trials, but it was not described [22,25]. One study did not indicate if the analyses were done on an ITT basis, so it is difficult to determine if the participants remained randomized throughout the study [23]. Two trials had multiple study centres where it was not possible to determine if the outcomes were comparable across all sites [22,23]. In one prospective cohort study, several baseline demographic and clinical characteristics differed among the patient groups, which may be associated with the measured outcomes [24]. For example, patients who received CABG surgery were older, were more likely to be male or diabetic, have a previous history of MI or increased disease severity versus patients with PTCA [24]. Patients with coronary stents also were more likely to have a positive MI history, a three-vessel CAD, and left anterior descending or stenosis greater than 50% in the right coronary artery compared with patients with PTCA [24].

### Data analysis and synthesis

#### Adverse events

No in-hospital adverse events, such as death, acute stent thrombosis, myocardial infarction or revascularization, were reported to have occurred due to a complication of balloon angioplasty [26]. One case of in-hospital myocardial infarction was reported in one study for a patient with angina, who was treated with a standard angioplasty balloon (1/106; 0.9%) [23]. Furthermore, two patients suffered from hematoma at a puncture site (2/106; 1.9%), and from contrast reaction (2/106; 1.9%). In the same study, no in-hospital major acute coronary events were found with patients who were treated with carbon-coated stents (0/116; 0%). One patient, however, experienced an aneurysm at a puncture site with the use of a stent [23]. In a single-arm study, the frequency of coronary perforation was the highest with the application of coronary balloon angioplasty (13/21; 61.9%) compared with guidewire manipulation (5/21; 20.8%) and coronary stenting (3/21; 14.3%) [28]. Coronary perforation is a rare but sometimes fatal complication associated with PCI [33]. Another single-arm study, which included only patients with a coronary perforation, found that there were more patients with a perforation with the use of intracoronary compliant balloon catheters (15/56; 26.8%) versus intracoronary noncompliant balloon catheters (13/56; 23.2%) and cutting balloons (4/56; 7.1%); however, there was no data on the total number of patients using each type of balloon [28]. Among patients who experienced a grade III coronary perforation, Shimony *et al*. found that the use of wires (30/57; 53%) was associated with the greatest frequency of coronary perforation compared with balloon catheters (15/57; 26%) and stents (12/57; 21%) [27]. This study only included patients with perforations, therefore, no data on the number of patients treated with each device was recorded. Long-term mortality was 22.1% (53/240) among patients who underwent CABG versus those with PTCA (19.9%; 95/478) and those with coronary stents (16.2%; 45/278) [24]. A greater proportion of patients with PTCA experienced major adverse cardiac events (66.3%; 317/478) versus those with coronary stents (49.6%; 138/278), followed by CABG (33.3%; 80/240) [24]. Conclusions regarding differences in rates in this study are impossible because of a lack of randomization.

#### Procedure complications

There were no procedure complications associated with balloon or balloon-coated catheters reported in two studies [22,29]. Procedural success occurred in 96.4% (27/28) of cases with paclitaxel-coated balloon catheters versus 100% (29/29) with Taxus stents [25]. One study reported occurrences of failure with the use of Stringray balloons. For instance, failure to advance into the proper position (2/14; 14.3%), to advance after a re-entry attempt (3/10; 30%) and a re-entry puncture as a result of loss of distal contrast filling (2/10; 20%) were reported [21].

#### Device malfunctions

One study indicated that there were no balloon ruptures or failure of delivery with the application of a 1.25-mm-diameter angioplasty balloon among patients with CAD [29]. Occurrences of device malfunctions were not mentioned in the remaining studies.

## Discussion

### Summary of evidence

Our systematic review included 11 studies that reported in-hospital adverse events, procedure complications and device malfunctions with the use of PTCA catheters in patients with CAD. In-hospital adverse events reported were individual cases of myocardial infarction and hematoma. In studies of patients with coronary perforation, more patients with balloon angioplasty were identified compared with patients who required stenting; however, these studies neglected to evaluate the at-risk population. As more patients undergo angioplasty than stenting, then the comparative risk is uncertain. In these studies [27,28], there were conflicting results regarding balloon angioplasty and guidewires. Since the interventions were not described in the studies, and study design and sample differed, it was difficult to determine the discrepancies in the findings between the studies. Procedure complications and device malfunctions were reported rarely and when they were they occurred infrequently. One review found that balloon-only PTCA was associated with a greater risk of angiographic restenosis versus bare-metal and drug-eluting stents [34]. Long-term adverse events reported in one study included mortality and MACE [24].

Our study highlights that a systematic review of the published and grey literature as a method to identify potential safety issues or ruptures associated with the use of PTCA catheters in patients with CAD is inadequate compared with the number of adverse reports submitted to the FDA in 2011 (1,942 adverse events in 2011). In most studies, the objectives were not to monitor the long-term safety aspects on the use of PTCA catheters in clinical practice. For instance, many studies ranged from 6 to 18 months in length, and their study populations were less than 250 patients. Even for short-term studies, the reporting of procedure- and device-related adverse events was inadequate. For example, several studies did not identify a denominator, so a hazard or risk ratio could not be determined. As the main objective of most selected studies was not to determine the incidents, adverse events or malfunctions associated with the use of PTCA catheters, it is uncertain that contacting the principal investigators of the published studies would have yielded additional insights with regards to their safety. Furthermore, there was no single classification system for complications, making it difficult to compare or aggregate adverse event types and risk across studies. Although the majority of the adverse event reports associated with the use of PTCA catheters submitted to the FDA were related to material rupture (data not reported), none of the selected studies reported any device malfunction when used in their clinical setting. Furthermore, the patient outcomes for 1,662 adverse event reports submitted to the FDA remained unknown. This lack of information precludes us from a comparison analysis between these outcomes and patient outcomes reported in the selected studies.

Another limitation is the lack of standard reporting of device brands and models. PTCA catheter brands and models were reported in only four studies [21,23,29,30]. Medical devices do not have an assigned unique identifier. Consequently, it is impossible to identify the specific device used in a patient if an adverse event occurs after the fact. Furthermore, none of the retrospective studies described the registries or existing surveillance programs in their institutions. Details of the structures used for data collection, intended use of the data and subsequent approaches to improve patient care would offer clarification on PMS in clinical practice. Important considerations in the development of research methodologies to identify the risk of adverse events and device malfunctions include product lifecycle, the learning curve of the device operator and the use of a device external to the original indication [35].

RCTs are considered to be the gold standard in the medical literature. Numerous disadvantages, however, exist with the sole use of clinical trials to assess the safety and effectiveness of medical devices. They are as follows: inadequate sample size to detect rare adverse events, short follow-up period, reduced generalizability of findings due to strict exclusion criteria, difficulties with maintaining blinding and allocation concealment, and high costs associated with designing many clinical trials for technologies that are evolving rapidly [36]. Normand *et al*. proposed a framework that combines pre-market and post-market data to measure the performance of medical devices [36]. To illustrate the proposed methods, a Bayesian hierarchical method was employed to combine clinical scores and outcomes from three studies using a RCT and observational designs on hip arthroplasty. The authors concluded that this method allows the performance data of medical devices to be monitored throughout the product lifecycle, increases transparency with explicit assumptions and is indicative of existing evidence gaps [36].

### Limitations

Our literature search strategy was limited to the published and grey literature of full-text reports available in the past five years, to determine the impact of the reclassification of PTCA catheters from a class III to class II risk level by the FDA in 2011 and to identify studies relevant to current clinical practice. Studies were also restricted to English and French publications due to limited resources and time restrictions. Although Morrison *et al*. found no systematic bias when English-language restrictions were imposed in systematic reviews, the authors of the current review acknowledge that some bias may still exist by imposing language restrictions in the literature search strategy as further research is required in this area [37]. It is unlikely that we would have found a sufficient number of studies in other languages to change our conclusion about the gap between the number of reports in the published medical and grey literature and the number of reports from the FDA as the gap is significant. In addition, the inclusion criteria were defined by the published and grey literature. According to the results of the systematic review, it is a challenge to estimate accurately the extent of adverse events and device malfunctions associated with the use of PTCA in patients with CAD based solely on the available published and grey literature. As our primary study objective was to review the PMS studies in the medical and grey literature published between 2007 and 2012, we did not contact the authors of the included studies to obtain additional details. The limited information available in some of these studies is indicative of the inadequate reporting of PMS associated with the use of PTCA catheters in patients with CAD in the medical and grey literature. Doshi *et al*. suggested that complete and anonymized clinical study reports would provide supplemental evidence that is not available in publications, partly due to word limits imposed by biomedical journals [38]. For instance, one published trial on oseltamivir reported no adverse events, while the clinical study report listed three that may have been associated with its use [38]. On the other hand, manufacturers are not required to conduct a clinical trial to demonstrate the safety and efficacy of a medical device for pre-market approval.

### Directions for future research

Medical devices that pose an increased risk of harm in patients could be an important indicator for the discontinuation of their use in patients. Our systematic review, however, illustrates that the volume and quality of PMS studies associated with the PTCA catheters in patients with CAD are low in the published and grey literature, and are not useful sources of information for decisions on safety. The validity and feasibility of other methods, such as the use of both published and unpublished data, that can inform the risk of the use of a medical device warrant further exploration.

Since clinical trials are not required for medical devices for pre-market approval, databases and surveillance systems for reporting adverse events associated with medical devices may provide some insight that is unavailable in published studies. Passive surveillance systems, such as the MAUDE database [39], are considered to underreport adverse events. Reasons for underreporting include the inability to link an adverse event to a specific device, lack of awareness of the reporting systems, a lack of an obligation to report adverse events and concerns with liability issues [36]. Another concern with surveillance systems is the challenge in accurately detecting the cause of the adverse events. Further investigations are required to improve current surveillance systems. The FDA launched MedSun, a Medical Product Safety Network, in 2002. To date, trained representatives from over 350 health-care facilities, report adverse events to MedSun that occurred in their facility resulting from the use of a medical device [40]. One challenge that remains is capturing the number of devices used in practice, which would give a better understanding of the rate of related adverse events [41]. The comprehensiveness of the database and its impact in risk management merit further investigation.

New initiatives, such as the Medical Device Epidemiology Network, spearheaded by the FDA Center for Devices and Radiologic Health, aim to develop and advance research methods to improve the accuracy and volume of evidence on the safety and effectiveness of medical devices through collaborations with academic institutions. At the time of writing this review, the network was not fully operational [42], so its impact both on PMS and in clinical practice cannot be assessed yet. Future studies may examine innovative statistical methods to combine information from diverse data sources and study designs to assess the safety and effectiveness of medical devices, such as PTCA catheters, accurately.

## Conclusions

Our systematic review included 11 studies on PMS of PTCA catheters in patients with CAD. The study designs included single- and multiple-arm prospective and retrospective studies, non-blinded RCTs and case–control studies. Their internal validity was generally low. The reported in-hospital and long-term adverse events involved coronary perforations, MACE and mortality. There were limited reports of adverse events common in clinical practice such as procedure failures, distal vessel occlusion due to embolization and puncture site complications. One study did not observe any device malfunctions, and the procedural complications reported in two studies did not appear to impact patient care severely. Given the volume of adverse events reported to the FDA since 2008, it is unlikely that the published and grey literature represent the full spectrum of device malfunctions and adverse events caused by the use of PTCA catheters. Future studies may explore the strengths and limitations of PMS databases administered by regulatory authorities. Innovative study designs and statistical methods to measure the safety and effectiveness of medical devices require further exploration.

## Abbreviations

CAD: Coronary artery disease; CP: Coronary artery perforation; CS: Coronary stent; CTO: Coronary total occlusion; CTR: Clinical trial registration; DES: Drug eluting stent; FDA: US Food and Drug Administration; ISR: In-stent restenosis; ITT: Intention-to-treat; MACE: Major adverse cardiac event; MeSH: Medical subject headings; MI: Myocardial infarction; NR: Not reported; NSTEMI: Non-ST elevation myocardial infarction; PCI: Percutaneous coronary intervention; PMA: Pre-market approval; PMS: Post-marketing surveillance; POBA: Plain old balloon angioplasty; PRISMA: Preferred reporting items for systematic reviews and meta-analyses; PTCA: Percutaneous transluminal coronary angioplasty; RCT: Randomized clinical trial; TVR: Target vessel revascularization.

## Competing interests

The authors declare that they have no competing interests.

## Authors’ contributions

JP led the systematic review and preparation of the manuscript. AJF contributed to and reviewed the draft versions of the manuscript. KC participated in the systematic review and reviewed the draft versions of the manuscript. DR developed and ran the literature search and reviewed draft versions the manuscript. All authors read and approved the final manuscript.

## Supplementary Material

Additional file 1: Table S1 Literature search strategy. Click here for file
