# Supplementary material for: Post-marketing surveillance in the published medical and grey literature for percutaneous transluminal coronary angioplasty catheters: a systematic review
Source: Syst Rev. 2013 Oct 10;2:94. doi: 10.1186/2046-4053-2-94 (PMC3853687; doi:10.1186/2046-4053-2-94)
Supplement: Additional file 1: Table S1 — Literature search strategy. [file 2046-4053-2-94-S1.docx]

**SUPPLEMENTAL TABLE 1: LITERATURE SEARCH STRATEGY**

**Cochrane Central Register of Controlled Trials**

**Ovid MEDLINE**

| **#** | **Searches** |
| --- | --- |
| 1 | Angioplasty, Balloon, Coronary/ |
| 2 | exp Heart Catheterization/is |
| 3 | ((cutting or scoring) adj6 catheter*).ti,ab. |
| 4 | "Percutaneous transluminal coronary angioplast*".ti,ab. |
| 5 | PTCA.ti. |
| 6 | PTCA.ab. /freq=2 |
| 7 | exp Heart Catheterization/ or ((coronary or heart or angioplasty) adj3 catheter*).ti. |
| 8 | ((coronary or heart or angioplasty) adj3 catheter*).ab. /freq=2 |
| 9 | 7 or 8 |
| 10 | (angioplasty or angioplasties).ti. or exp Angioplasty/ |
| 11 | (angioplasty or angioplasties).ab. /freq=2 |
| 12 | 10 or 11 |
| 13 | 9 and 12 |
| 14 | 1 or 2 or 3 or 4 or 5 or 6 or 13 |
| 15 | Safety/ |
| 16 | Safety Management/ |
| 17 | Equipment Safety.sh. |
| 18 | (Equipment Failure or Equipment Failure Analysis or Intrauterine Device Expulsion or Prosthesis Failure).sh. |
| 19 | Consumer Product Safety/ |
| 20 | "Product Recalls and Withdrawals"/ |
| 21 | Medical Device Recalls/ |
| 22 | "Safety-Based Medical Device Withdrawals"/ |
| 23 | Product Surveillance, Postmarketing/ |
| 24 | Clinical Trials, Phase IV as Topic/ |
| 25 | Clinical Trial, Phase IV.pt. |
| 26 | exp Postoperative complications/ |
| 27 | exp Intraoperative complications/ |
| 28 | exp Poisoning/ |
| 29 | exp side effect/ |
| 30 | exp postmarketing surveillance/ |
| 31 | exp phase 4 clinical trial/ |
| 32 | exp postoperative complication/ |
| 33 | exp peroperative complication/ |
| 34 | exp intoxication/ |
| 35 | "Side Effects (Treatment)"/ |
| 36 | (Hazard* or defect* or misuse* or failure* or malfunction* or recall* or withdr?w*).ti. |
| 37 | (Safe or safety or unsafe).ti. |
| 38 | Side effect*.ti. |
| 39 | (Adverse or undesirable or harm* or injurious or risk or risks or reaction* or toxic or toxicit* or toxologic* or complication* or noxious or tolerability or poison* or intoxication or warning*).ti. |
| 40 | (Hazard* or defect* or misuse* or failure* or malfunction* or recall* or withdr?w*).ab. /freq=2 |
| 41 | ((Adverse or undesirable or harm* or toxic or injurious or serious) adj3 (effect* or reaction* or event* or outcome* or incident*)).ab. /freq=2 |
| 42 | (Side effect* or Safety or unsafe).ab. /freq=2 |
| 43 | or/15-42 |
| 44 | ("post marketing" or postmarketing or postmarket).ti,ab. |
| 45 | ((postoperative or "post operative") adj3 (complication* or surveillance or outcome* or analysis or stud*)).ti,ab. |
| 46 | (clinical adj (outcome* or effectiveness)).ti. |
| 47 | (clinical adj (outcome* or effectiveness)).ab. /freq=2 |
| 48 | 43 or 44 or 45 or 46 or 47 |
| 49 | 14 and 48 |
| 50 | exp Heart Catheterization/ae |
| 51 | 49 or 50 |
| 52 | exp animals/ |
| 53 | exp animal experimentation/ |
| 54 | exp models animal/ |
| 55 | exp animal experiment/ |
| 56 | nonhuman/ |
| 57 | exp vertebrate/ |
| 58 | animal.po. |
| 59 | or/52-58 |
| 60 | exp humans/ |
| 61 | exp human experiment/ |
| 62 | human.po. |
| 63 | or/60-62 |
| 64 | 59 not 63 |
| 65 | (comment or newspaper article or editorial or letter or note).pt. |
| 66 | case series.ti,ab. |
| 67 | case reports.pt. |
| 68 | (case adj3 (report or reports or study or studies or histories)).ti,ab. |
| 69 | organizational case studies/ |
| 70 | 64 or 65 or 66 or 67 or 68 or 69 |
| 71 | 51 not 70 |

**Embase**

| **#** | **Searches** |
| --- | --- |
| 1 | percutaneous transluminal angioplasty/ |
| 2 | transluminal coronary angioplasty/ |
| 3 | *heart catheterization/ |
| 4 | ((cutting or scoring) adj6 catheter*).ti,ab. |
| 5 | "Percutaneous transluminal coronary angioplast*".ti,ab. |
| 6 | PTCA.ti. |
| 7 | PTCA.ab. /freq=2 |
| 8 | exp *Heart Catheterization/ or ((coronary or heart or angioplasty) adj3 catheter*).ti. |
| 9 | ((coronary or heart or angioplasty) adj3 catheter*).ab. /freq=2 |
| 10 | 8 or 9 |
| 11 | exp *Angioplasty/ or (angioplasty or angioplasties).ti. |
| 12 | (angioplasty or angioplasties).ab. /freq=2 |
| 13 | 11 or 12 |
| 14 | 10 and 13 |
| 15 | 1 or 2 or 3 or 4 or 5 or 6 or 7 or 14 |
| 16 | Safety/ |
| 17 | Safety Management/ |
| 18 | Equipment Safety.sh. |
| 19 | (Equipment Failure or Equipment Failure Analysis or Intrauterine Device Expulsion or Prosthesis Failure).sh. |
| 20 | Consumer Product Safety/ |
| 21 | "Product Recalls and Withdrawals"/ |
| 22 | Medical Device Recalls/ |
| 23 | "Safety-Based Medical Device Withdrawals"/ |
| 24 | Product Surveillance, Postmarketing/ |
| 25 | Clinical Trials, Phase IV as Topic/ |
| 26 | Clinical Trial, Phase IV.pt. |
| 27 | exp Postoperative complications/ |
| 28 | exp Intraoperative complications/ |
| 29 | exp Poisoning/ |
| 30 | exp side effect/ |
| 31 | exp postmarketing surveillance/ |
| 32 | exp phase 4 clinical trial/ |
| 33 | exp postoperative complication/ |
| 34 | exp peroperative complication/ |
| 35 | exp intoxication/ |
| 36 | "Side Effects (Treatment)"/ |
| 37 | (Hazard* or defect* or misuse* or failure* or malfunction* or recall* or withdr?w*).ti. |
| 38 | (Safe or safety or unsafe).ti. |
| 39 | Side effect*.ti. |
| 40 | (Adverse or undesirable or harm* or injurious or risk or risks or reaction* or toxic or toxicit* or toxologic* or complication* or noxious or tolerability or poison* or intoxication or warning*).ti. |
| 41 | (Hazard* or defect* or misuse* or failure* or malfunction* or recall* or withdr?w*).ab. /freq=2 |
| 42 | ((Adverse or undesirable or harm* or toxic or injurious or serious) adj3 (effect* or reaction* or event* or outcome* or incident*)).ab. /freq=2 |
| 43 | (Side effect* or Safety or unsafe).ab. /freq=2 |
| 44 | or/16-43 |
| 45 | ("post marketing" or postmarketing or postmarket).ti,ab. |
| 46 | ((postoperative or "post operative") adj3 (complication* or surveillance or outcome* or analysis or stud*)).ti,ab. |
| 47 | (clinical adj (outcome* or effectiveness)).ti. |
| 48 | (clinical adj (outcome* or effectiveness)).ab. /freq=2 |
| 49 | 44 or 45 or 46 or 47 or 48 |
| 50 | 15 and 49 |
| 51 | *heart catheterization/ae, co |
| 52 | 50 or 51 |
| 53 | exp animals/ |
| 54 | exp animal experimentation/ |
| 55 | exp models animal/ |
| 56 | exp animal experiment/ |
| 57 | nonhuman/ |
| 58 | exp vertebrate/ |
| 59 | animal.po. |
| 60 | or/53-59 |
| 61 | exp humans/ |
| 62 | exp human experiment/ |
| 63 | human.po. |
| 64 | or/61-63 |
| 65 | 60 not 64 |
| 66 | (comment or newspaper article or editorial or letter or note).pt. |
| 67 | case series.ti,ab. |
| 68 | (case adj3 (report or reports or study or studies or histories)).ti,ab. |
| 69 | case study/ or case report/ |
| 70 | conference abstract.pt. |
| 71 | 65 or 66 or 67 or 68 or 69 or 70 |
| 72 | 52 not 71 |

**PubMed in-process**

| **Search** | **Query** |
| --- | --- |
| #1 | Search ((cutting[tiab] OR scoring[tiab]) AND catheter*[tiab]) |
| #2 | Search Percutaneous[tiab] AND transluminal[tiab] AND coronary[tiab] AND angioplast*[tiab] |
| #3 | Search PTCA[tiab] |
| #4 | Search Angioplasty[tiab] AND catheter[tiab] AND Coronary[tiab] |
| #5 | Search ((coronary[tiab] OR heart[tiab] OR angioplasty[tiab]) AND catheter*[tiab]) |
| #6 | Search (angioplasty[tiab] OR angioplasties[tiab]) |
| #7 | Search #5 AND #6 |
| #8 | Search #1 OR #2 OR #3 OR #4 OR #7 |
| #9 | Search Safety[mh] OR Safety Management[mh] OR Equipment Safety[mh] OR Equipment Failure[mh] OR Equipment Failure Analysis[mh] OR Consumer Product Safety[mh] OR “Product Recalls and Withdrawals”[mh] OR Medical Device Recalls[mh] OR Safety-Based Medical Device Withdrawals[mh] OR Product Surveillance, Postmarketing[mh] OR Clinical Trials, Phase IV as Topic[mh] OR Clinical Trial, Phase IV[pt] OR Postoperative Complications[mh] OR Intraoperative Complications[mh] OR Poisoning[mh] OR adverse[ti] OR undesirable[ti] OR harm*[ti] OR injurious[ti] OR risk[ti] OR risks[ti] OR reaction*[ti] OR toxic[ti] OR toxicit*[ti] OR toxologic*[ti] OR complication*[ti] OR noxious[ti] OR tolerability[ti] OR poison*[ti] OR intoxication[ti] OR hazard*[ti] OR defect*[ti] OR misuse*[ti] OR failure*[ti] OR malfunction*[ti] OR recall*[ti] OR withdrew[ti] OR withdrawn[ti] OR withdrawal*[ti] OR safe[ti] OR safety[ti] OR unsafe[ti] OR Side effect*[ti] OR warning*[ti] |
| #10 | Search (“post marketing”[tiab] OR postmarketing[tiab] OR postmarket[tiab]) |
| #11 | Search ((postoperative[tiab] OR “post operative”[tiab]) AND (complication[tiab] OR complications[tiab] OR surveillance[tiab] OR outcome[tiab] OR outcomes[tiab] OR analysis[tiab] or study[tiab] OR studies[tiab])) |
| #12 | Search clinical[tiab] AND (outcome[tiab] OR outcomes[tiab] OR effectiveness[tiab]) |
| #13 | Search #9 OR #10 OR #11 OR #12 |
| #14 | Search #8 AND #13 |
| #15 | Search case series[tiab] |
| #16 | Search case reports[sb] |
| #17 | Search (case[tiab] AND (report[tiab] OR reports[tiab] OR study[tiab] OR studies[tiab] OR histories[tiab])) |
| #18 | Search #15 OR #16 OR #17 |
| #19 | Search #14 NOT #18 |
| #20 | Search ((Animals[MESH] OR Animal Experimentation[MESH] OR "Models, Animal"[ MESH] OR animal*[tiab] OR nonhuman[tiab] OR non human[tiab] or rat[tiab] OR rats[tiab] OR mouse[tiab] OR mice[tiab] OR rabbit[tiab] OR rabbits[tiab] OR pig[tiab] OR pigs[tiab] OR porcine[tiab] OR swine[tiab] OR dog[tiab] OR dogs[tiab] or hamster[tiab] OR hamsters[tiab] or fish[tiab] or chicken[tiab] OR chickens[tiab] or sheep[tiab]) NOT (Humans[MESH] OR human[tiab])) |
| #21 | Search editorial[pt] OR comment[pt] OR letter[pt] OR newspaper article[pt] |
| #22 | Search #20 OR #21 |
| #23 | Search #19 NOT #22 |
| #24 | Search publisher[sb] |
| #25 | Search #23 AND #24 |
